# Exogenous Ubiquitin Differentially Modulates the Phenotype and Function of M1 and M2 Macrophages

**DOI:** 10.3390/cells14120879

**Published:** 2025-06-11

**Authors:** Paige L. Shook, Hui Wang-Heaton, Jared L. Casteel, Suman Dalal, Mahipal Singh, Valentin Yakubenko, Krishna Singh

**Affiliations:** 1 Department of Biomedical Sciences, James H. Quillen College of Medicine, East Tennessee State University, Johnson City, TN 37614, USA; shook.paige@mayo.edu (P.L.S.); wangh1@etsu.edu (H.W.-H.); casteelj@etsu.edu (J.L.C.); singhm@etsu.edu (M.S.); yakubenko@etsu.edu (V.Y.); 2 Department of Biomedical Health Sciences, College of Public Health, East Tennessee State University, Johnson City, TN 37614, USA; dalal@etsu.edu; 3 Center of Excellence for Inflammation, Infectious Disease and Immunity, East Tennessee State University, Johnson City, TN 37614, USA; 4 James H. Quillen Veterans Affairs Medical Center, East Tennessee State University, Johnson City, TN 37614, USA

**Keywords:** Ubiquitin, macrophage, polarization, inflammation, heart

## Abstract

Background: Unresolved inflammation is a major predictor of heart failure following myocardial infarction. Exogenous ubiquitin (eUB) is shown to decrease inflammatory response and confer cardioprotection in mice 3 days post-ischemia/reperfusion (I/R) injury. Here, we hypothesized that eUB differentially modulates the phenotype and function of M1 and M2 macrophages. Methods and Results: Peritoneal macrophages, pretreated with UB for 30 min, were exposed to IFN-γ (M1 polarization) or IL-4 (M2 polarization) for 72 h. Cytokine/chemokine levels were measured in conditioned media, while cells were used for functional and biochemical assays. eUB reduced TNF-α secretion in M1, and TNF-α and IL-10 secretion in M2 macrophages. eUB induced cytoskeletal reorganization and reduced surface area in M1 macrophages. eUB enhanced M1 migration; however, it decreased M2 macrophage migration and efferocytosis. It decreased STAT1 and FAK phosphorylation in M1, while increasing STAT6 and FAK phosphorylation in M2 macrophages. Total protein ubiquitination remained unchanged. In non-activated macrophages, eUB altered morphology, suppressed IL-1β, IL-2, and IL-5 secretion, and enhanced efferocytosis. Conclusion: eUB modulates macrophage polarization, reduces pro-inflammatory cytokine secretion, and alters functional parameters and intracellular signaling. These effects may contribute to the cardioprotective potential of eUB 3 days post-I/R injury.

## 1. Introduction

Cardiovascular disease is one of the leading causes of death worldwide, with myocardial infarction (MI) being one of the most common implications [1]. MI is an ischemic injury to the heart resulting in myocardial cell death due to blocked blood flow-induced hypoxemia [2]. Inflammation post-MI is critical to cardiac repair; however, dysregulated inflammation is considered as a predictor of heart failure [3,4]. Macrophages are essential in the innate immune response post-MI. Cytokines and chemokines, produced post-MI, promote circulating monocyte migration and differentiation into macrophages in the infarct [5,6]. Various mediators in the tissue microenvironment dictate the polarization of these macrophages towards a pro-inflammatory (M1) or anti-inflammatory (M2) phenotype. During the acute inflammatory phase (1 day post-MI), the pro-inflammatory phenotype dominates, and these macrophages produce inflammatory cytokines [7]. As myocardial remodeling progresses (3–7 days post-MI), the macrophage phenotype becomes more anti-inflammatory, producing mediators that regulate extracellular matrix remodeling [7]. Although M1 and M2 phenotypes are not indicative of the entire range of macrophage polarization in vivo, they are used in this study to represent the pro-inflammatory and anti-inflammatory phenotypes observed during myocardial wound healing response post-MI.

Ubiquitin (UB), a highly evolutionarily conserved small-molecular-weight protein, is present in eukaryotic cells. The primary intracellular function of UB is to tag proteins for degradation via the ubiquitin–proteasome system (UPS) [8,9]. UB is also present in body fluids such as urine, serum, and cerebrospinal fluid. Increased extracellular levels of UB are observed in multiple disease states, including coronary artery disease [10]. In the heart, exogenous UB (eUB) is shown to have immunomodulatory as well as cardioprotective effects post-ischemia/reperfusion (I/R) injury in mice [10,11,12]. eUB has also been shown to affect immune cells. In THP-1 monocytes, eUB treatment has been associated with chemotactic effects [13]. In non-activated macrophages differentiated from THP-1 monocytes, eUB promoted M2 macrophage polarization [14]. 

Here, we tested the hypothesis that eUB decreases secretory levels of inflammatory cytokines and differentially affects the phenotype and function of M1- and M2-polarized macrophages. This study used macrophages isolated from the peritoneal cavity, as the peritoneal cavity provides sufficient number of macrophages, specifically following thioglycolate injection, to perform functional and biochemical assays. Peritoneal macrophages are also considered to be more mature and stable with respect to their phenotype and function when compared to the macrophages isolated from bone marrow or spleen [15]. The data presented here suggests that eUB reduces secretory levels of cytokines/chemokines in M1- and M2-polarized macrophages, induces phenotypic alterations, enhances migration of M1-polarized macrophages, and reduces efferocytosis and migration of M2-polarized macrophages. The effects of eUB may occur via the alterations in the activation of STAT1, STAT6 and FAK. 

## 2. Materials and Methods

### 2.1. Animals

Male C57BL/6J mice, aged 8–12 weeks (22–27 g; Jackson Laboratory), were used for isolation of peritoneal macrophages. All experimental procedures were reviewed and approved by the East Tennessee State University Committee on Animal Care. The investigation conforms to the Guide for the Care and Use of Laboratory Animals published by the US National Institutes of Health (NIH Publication No. 85–23, revised 1996). 

### 2.2. Isolation of Peritoneal Macrophages

Mice were injected intraperitoneally with 1 mL of 4% thioglycolate. Following 72 h of injection, cells from the peritoneal cavity were harvested by lavage using 5 mL of sterile PBS. Cells were washed with sterile PBS followed by resuspension in Dulbecco’s modified Eagle’s medium (DMEM; Mediatech, Atlanta, USA) supplemented with 25 mM HEPES, 1.0% penicillin–streptomycin, and 10% fetal bovine serum (FBS). Cells were then transferred to either 60 mm (2 × 10^6^) or 6-well (1.5 × 10^6^) dishes and incubated at 37 °C in humidified air containing 5% CO_2_ atmosphere for 2 h. The cells were washed with sterile PBS to remove non-adherent cells. The media were replenished with antibiotic-supplemented DMEM containing heat-inactivated 1.0% charcoal-stripped FBS [16].

### 2.3. Macrophage Activation and Treatment

Macrophages were pretreated with eUB (20 ug/mL) for 30 min followed by treatment with IFN-γ (100 U/mL; for M1 polarization), or IL-4 (20 ng/mL; for M2 polarization) for 72 h. The adherent cells cultured for 72 h without any treatment were considered non-activated macrophages [17]. Culture media were refreshed with the respective treatments at the 48 h timepoint [16]. Cells were considered polarized following 72 h of treatment with either IFN-γ or IL-4, and were used for experiments at this timepoint. The experimental groups were control (CTL), IFN-γ, UB+IFN-γ, IL-4, UB+IL-4 and UB. 

### 2.4. Measurement of Cytokines/Chemokines Levels in the Conditioned Media

Conditioned media of peritoneal macrophages were collected 72 h post-polarization and centrifuged at 1000× g for 15 min. The supernatants were used to measure cytokines/chemokines levels using a Bio-Plex Pro Mouse Cytokine 8-Plex assay kit (Bio-Rad). The kit consisted of the following cytokines/chemokines: interleukin-1β (IL-1β), IL-2, IL-4, IL-5, IL-10, granulocyte–macrophage colony-stimulating factor (GM-CSF), interferon (IFN)-γ, and tumor necrosis factor (TNF)-α.

### 2.5. Cell Morphology

F-actin phalloidin fluorescent staining was used to monitor changes in macrophage morphology. Phalloidin-stained images were also used to quantify the cell spreading areas of polarized macrophages. For this, macrophages plated on glass coverslips were polarized for 72 h, then washed with sterile PBS and fixed with 4% paraformaldehyde (PFA). Cells were then permeabilized with 0.1% Triton X-100 and stained with Phalloidin-iFluor 555 (1:1000, ThermoFisher, Atlanta, GA, USA) to stain F-actin [16]. Images were acquired using the EVOS m7000 microscope (Melville, NY, USA). The Nikon NIS-Elements software was used to measure the cell surface area. 

### 2.6. Efferocytosis Assay

Macrophages, plated on a 6-well dish, were polarized for 72 h in the presence or absence of eUB. Neutrophils, isolated from the peritoneal cavity 4 h post-thioglycolate injection, were treated with PMA (phorbol-12-myristate-13-acetate; 30 ng/mL) and H_2_O_2_ (0.1 mM) to stimulate apoptosis/necrosis. Neutrophils were then labeled with PKH67 and incubated overnight with macrophages. Cells were washed twice with PBS, fixed with 4% PFA, and stained with anti-F4/80 antibodies (1:200, Santa Cruz Biotech, Dallas, TX, USA) to label macrophages. Nuclei were visualized using Hoechst 33258 (10 uM; Sigma, Saint Louis, MO, USA) staining. To quantify efferocytosis, images were acquired using the EVOS m7000 microscope. Efferocytosis was quantified as the percentage of macrophages with engulfed PKH67-labeled neutrophils using Nikon NIS-Elements software [18].

### 2.7. Two-Dimensional Migration Assay

Macrophages, plated on 6-well dishes, were polarized for 72 h in the presence or absence of eUB. At this point, a wound was created in the center of the confluent monolayer by removing cells using a sterile plastic pipette tip. Cells were washed twice with PBS to remove cellular debris, and cell culture media and treatments were replenished. Images of the wound area were acquired with brightfield imaging using an EVOS m7000 microscope at 0 and 4 h timepoints. The wound area recovered was quantified with Nikon NIS-Elements software as distance in µm traveled since the 0 h timepoint [19].

### 2.8. Western Blot Analysis

Macrophages, polarized for 72 h in the presence or absence of eUB, were lysed using lysis buffer (10 mM Tris-HCl pH7.5, 150 mM NaCl, 1% Triton X-100, 1 mM EDTA, 1 mM EGTA, 0.5% Nonidet P-40, 0.2 mM sodium orthovanadate, 1 mM phenylmethylsulphonyl fluoride). Protein concentration was measured using the Bradford assay (Bio-Rad Laboratories, Inc., Hercules, CA, USA). Equal amounts of proteins (10 µg) were resolved by 7.5% or 10% SDS-PAGE and transferred onto a PVDF membrane. The Pierce^™^ reversible total protein stain kit (#24585, Thermo Fisher Scientific, Atlanta, GA, USA) was used to achieve total protein staining. Membranes were blocked in 5% non-fat milk for 1 h at room temperature and incubated overnight at 4 °C with primary antibodies. Primary antibodies directed against p-Stat1 (Tyr 701; 1:1000; #9167), p-Stat6 (Tyr 641; 1:2000; #56554) and p-FAK (Tyr 397; 1:500; #3283) were purchased from Cells Signaling Technology (Danvers, MA, USA). Antibodies against UB (1:500; #sc-8017) and GAPDH (1:10,000; #sc-32233) were obtained from Santa Cruz Biotechnology (Dallas, TX, USA). The membranes were washed and incubated with appropriate secondary antibodies. Membranes were stripped using Restore™ Western blot stripping buffer (#21059, Thermo Fisher Scientific, Atlanta, GA, USA), followed by a stripping test. Subsequently, the membranes were immunostained using antibodies against STAT1 (1:1000; #9172), STAT6 (1:2000; #5397) or FAK (1:1000; #3285), all of which were obtained from Cell Signaling Technology (Danvers, MA, USA). The LAS500 imaging system (GE Healthcare, Chicago, IL, USA) was used to document band (immune complexes) intensities. The band intensities were quantified using ImageQuant software. Western blot replicates are shown in Supplementary Materials (Figures S1–S6).

### 2.9. Statistical Analysis

Data are presented as mean ± SE. Statistical analysis was performed using one-way analysis of variance (ANOVA) followed by a post hoc Tukey’s test or a two-tailed Student’s *t*-test (GraphPad Prism version 10.4.2). Confidence intervals were calculated at the 95% level. Probability (p) values of <0.05 were considered statistically significant. The number (n) represents biological replicates. Confidence intervals and p values are shown in Supplementary Materials (Tables S1–S8).

## 3. Results

### 3.1. Exogenous UB Decreases Cytokine Levels in the Conditioned Media

**Figure 1:**
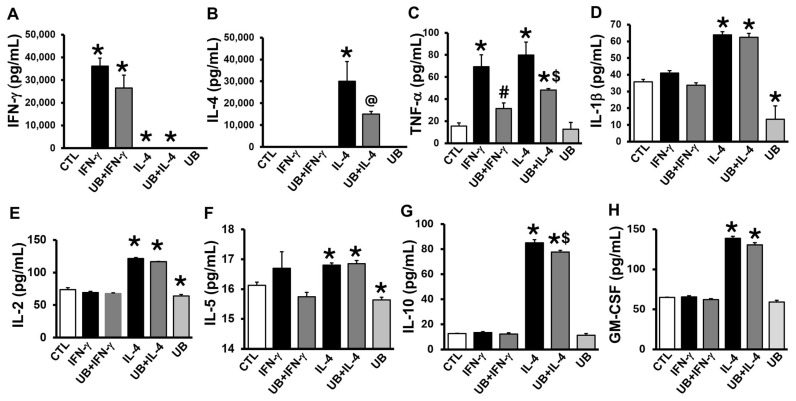
Exogenous UB decreases secretion of inflammatory cytokines in the conditioned media. Peritoneal macrophages, pretreated with UB (20 µg/mL) for 30 min, were treated with IFN-γ (100U/mL; M1 polarization) or IL-4 (20 ng/mL; M2 polarization) for 72 h. Levels of cytokines/chemokines were measured in the conditioned media using a Bio-Plex pro Mouse Cytokine 8-plex kit. Cytokines/chemokines are: (**A**) IFN-γ; (**B**) IL-4; (**C**) TNF-α; (**D**) IL-1β; (**E**) IL-2; (**F**) IL-5; (**G**) IL-10; (**H**) GM-CSF. Data were analyzed using one-way ANOVA followed by a post hoc Tukey’s test (* *p* < 0.05 vs. CTL, ^#^ *p* < 0.05 vs. IFN-γ; ^$^ *p* < 0.05 vs. IL-4;) and 2-tailed Student’s *t*-test (^@^ *p* < 0.05 vs. CTL); n = 4–5. The comparison groups were CTL, IFN-γ, UB+IFN-γ, and UB; and CTL, IL-4, UB+IL-4, and UB.

Measurement of cytokine levels using an 8-plex assay showed that treatment with IFN-γ and IL-4 dramatically increased IFN-γ and IL-4 levels in the conditioned media, respectively, confirming the validity of the assay (Figure 1A,B). In addition, M1 polarization using IFN-γ increased levels of TNF-α (Figure 1C), while M2 polarization using IL-4 increased levels of IFN-γ, TNF-α, IL-1β, IL-2, IL-5, IL-10, and GM-CSF versus control (Figure 1). eUB decreased IFN-γ-mediated increase in TNF-α (Figure 1C) and IL-4-mediated increase in TNF-α and IL-10 (Figure 1C,G) levels. In addition, levels of 1L-1β, IL-2 and IL-5 were lower in eUB-treated non-activated macrophages versus the control (Figure 1D–F).

### 3.2. Exogenous UB Modulates Phenotype of Macrophages

**Figure 2:**
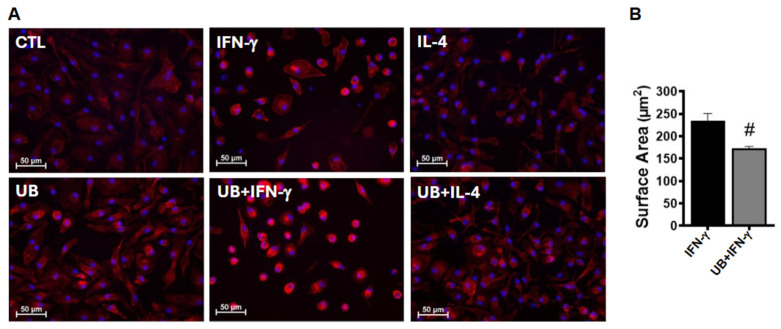
Exogenous UB modulates the phenotype and cell surface area of macrophages. Peritoneal macrophages, pretreated with UB (20 µg/mL) for 30 min, were treated with IFN-γ (100 U/mL; M1 polarization) or IL-4 (20 ng/mL; M2 polarization) for 72 h. Macrophages were stained by Phalloidin-iFluor 555 (F-actin staining; red) and Hoechst 33258 (nuclear staining; blue). Images were acquired using the EVOS m7000 microscope (**A**) and cell surface area was quantified using NIS-Elements software (**B**). Data were analyzed using a 2-tailed Student’s *t*-test. ^#^ *p* < 0.05 vs. IFN-γ; n = 3.

Macrophages, which are known to be heterogeneous cells, are capable of changing their morphology depending on environmental and molecular cues [20]. Actin polymerization and cytoskeletal reorganization induce morphological changes, affecting cell spreading and polarization of macrophages [21]. Analysis of F-actin-stained images showed that unpolarized control cells exhibit flat, elongated spindle-like morphology with the presence of few rounded cells. M1-polarized macrophages mostly acquired round, flat morphology with fewer projections. M1-polarized macrophages treated with eUB appeared smaller (Figure 2A). Analysis of F-actin-stained images showed a significant decrease in cell surface area in UB+IFN-γ group versus IFN-γ (Figure 2B). On the other hand, M2-polarized macrophages appeared bigger with elongated projections versus the control, with no difference in cell surface area between IL-4- and UB+IL-4-treated groups. eUB treated non-activated macrophages also acquired flat, elongated, and branched morphology versus control. In general, UB-treated groups exhibited prominent F-actin staining versus their respective control counterparts (Figure 2A). 

### 3.3. Exogenous UB Decreases M2-Polarized Macrophage Efferocytosis

**Figure 3:**
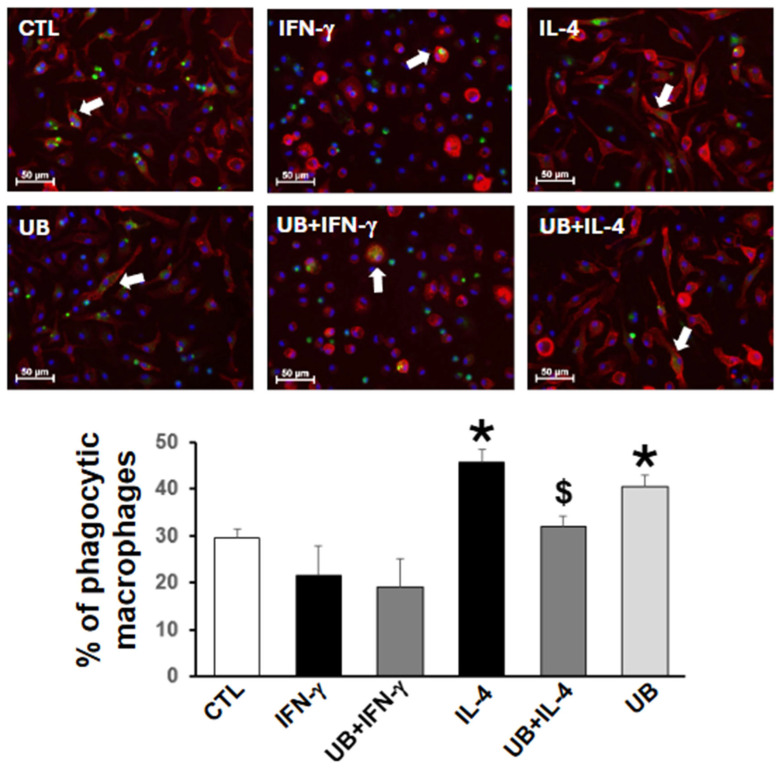
Exogenous UB affects efferocytosis of macrophages. Peritoneal macrophages, pretreated with UB (20 µg/mL) for 30 min, were treated with IFN-γ (100U/mL; M1 polarization) or IL-4 (20 ng/mL; M2 polarization) for 72 h. Cells were then incubated overnight with PKH67-labeled apoptotic neutrophils (green) and then immune-stained with anti-F4/80 antibodies (red). Nuclei were stained using Hoechst (blue). Images, captured using EVOS m7000 microscope, were analyzed using Nikon-NIS Elements software. White arrows indicate macrophages with engulfed apoptotic neutrophils. Data were analyzed using one-way ANOVA followed by a post hoc Tukey’s test. * *p* < 0.05 vs. CTL; ^$^ *p* < 0.05 vs. IL-4; n = 3. The comparison groups were CTL, IFN-γ, UB+IFN-γ, and UB; and CTL, IL-4, UB+IL-4, and UB.

Efferocytosis refers to the phagocytosis of apoptotic cells. Removal of apoptotic cells from the infarct region of the myocardium is an important function of macrophages post-MI [22,23]. Analysis of efferocytosis revealed that M1 polarization alone or in the presence of eUB has no effect on the percentage of efferocytosis versus the control (Figure 3). M2 polarization significantly increased the percentage of efferocytosis versus control. However, M2-polarized macrophages treated with eUB showed a significant decrease in the percentage of efferocytosis. In non-activated macrophages, eUB also increased efferocytosis versus control (Figure 3).

### 3.4. Exogenous UB Modulates Macrophage Migration

**Figure 4:**
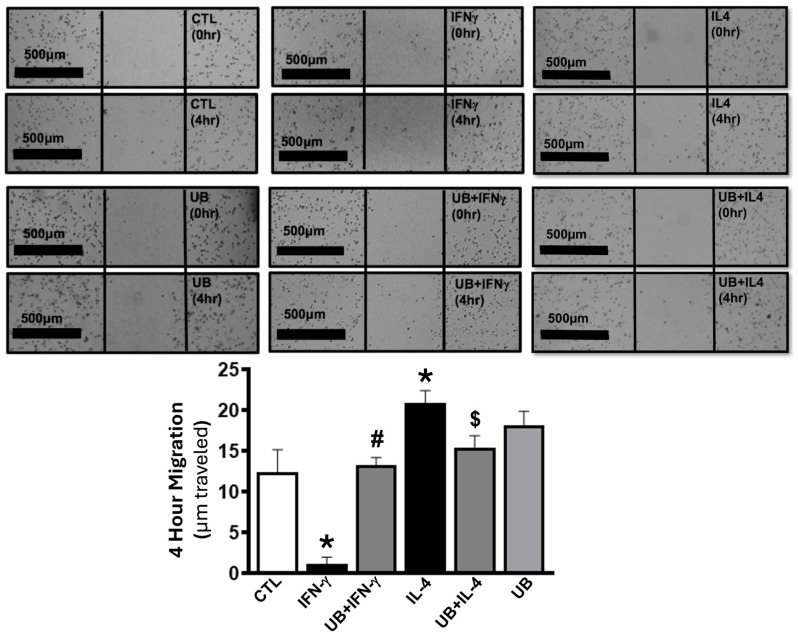
Exogenous UB modulates migration of M1- and M2-polarized macrophages. Peritoneal macrophages were treated with UB (20 µg/mL) for 30 min. The cells were then exposed to IFN-γ (100U/mL; M1 polarization) or IL-4 (20 ng/mL; M2 polarization) for 72 h, followed by creation of a wound using a sterile plastic pipette tip in the cell monolayer. Cell migration into the wound area was assessed 4 h after wound creation. To achieve this, images of the wound area were acquired using EVOS m7000 microscope. The captured images were analyzed with Nikon-NIS Elements software. The upper panels demonstrate the wound area at 0 and 4 h after wound creation. The lower panel shows the mean data as distance in µm traveled since 0 h timepoint. Data were analyzed using one-way ANOVA followed by a post hoc Tukey’s test (* *p* < 0.05 vs. CTL, ^#^ *p* < 0.05 vs. IFN-γ) and 2-tailed Student’s *t*-test (^$^ *p* < 0.05 vs. IL-4); n = 5. The comparison groups were CTL, IFN-γ, UB+IFN-γ, and UB; and CTL, IL-4, UB+IL-4, and UB.

Macrophage migration is a crucial process in inflammatory response post-MI [23]. The efflux of macrophages can contribute to the resolution of inflammation [24,25]. A 2D scratch assay demonstrated that M1 polarization inhibits the migration of cells into the wound area versus control. eUB treatment significantly enhanced the migration of M1-polarized macrophages (Figure 4). In contrast, M2 polarization significantly enhanced migration of macrophages into the wound versus control. This increase in migration in M2-polarized macrophages was significantly lower in the presence of eUB (Figure 4).

### 3.5. Exogenous UB Affects Intracellular Signaling

**Figure 5:**
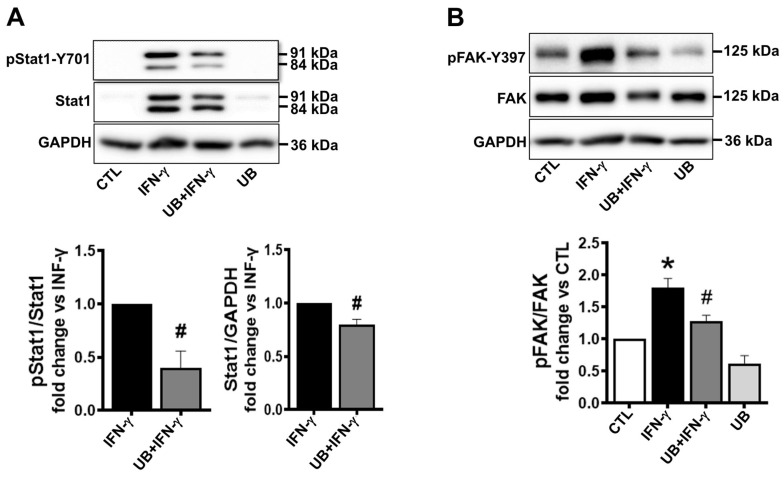
Exogenous UB decreases STAT1 and FAK activation in M1-polarized macrophages. Peritoneal macrophages, pretreated with UB (20 µg/mL) for 30 min, were treated with IFN-γ (100U/mL; M1 polarization) for 72 h. Cell lysates were analyzed by Western blot using anti-p-STAT1, STAT1, p-FAK, FAK, and GAPDH antibodies. After protein transfer, the membrane was cut into two portions. The upper portion, above the ~70 kDa marker, was probed for p-STAT1 and p-FAK, while the lower portion was probed for GAPDH. After stripping the upper portion, the membrane was probed for STAT1 and FAK. Thus, normalization for STAT1 and FAK was performed using the same GAPDH signal. (**A**) The upper panel exhibits immunoblotting for p-STAT1, STAT1 and GAPDH. (**B**) The upper panel exhibits immunoblotting for p-FAK, FAK and GAPDH. The lower panels (**A**,**B**) exhibit quantitative analyses normalized to STAT1, FAK or GAPDH. Data were analyzed using a 2-tailed Student’s *t* test or one-way ANOVA followed by a post hoc Tukey’s test. * *p* < 0.05 vs. CTL, ^#^ *p* < 0.05 vs. IFN-γ; n = 3.

IFN-γ is known to phosphorylate and activate the signal transducer and activator of transcription 1 (STAT1), which functions to regulate the transcription of many immune effector genes, including cytokines and chemokines [26,27]. Western blot analysis of cell lysates demonstrated low-level presence of STAT1 (84 and 91 kDa bands) in control samples and those treated with eUB alone with no detectable signal for p-STAT1. M1 polarization increased STAT1 expression and phosphorylation. However, STAT-1 expression and phosphorylation were significantly lower in eUB-treated M1-polarized cells versus IFN- γ (Figure 5A). 

Focal adhesion kinase (FAK) is involved in the formation of focal adhesions during cell migration [28]. Western blot analysis showed that M1 polarization increases FAK phosphorylation (Tyr397) by ~1.8-fold versus control. UB treatment significantly decreased the IFN-γ-mediated increase in FAK phosphorylation (Figure 5B). 

**Figure 6:**
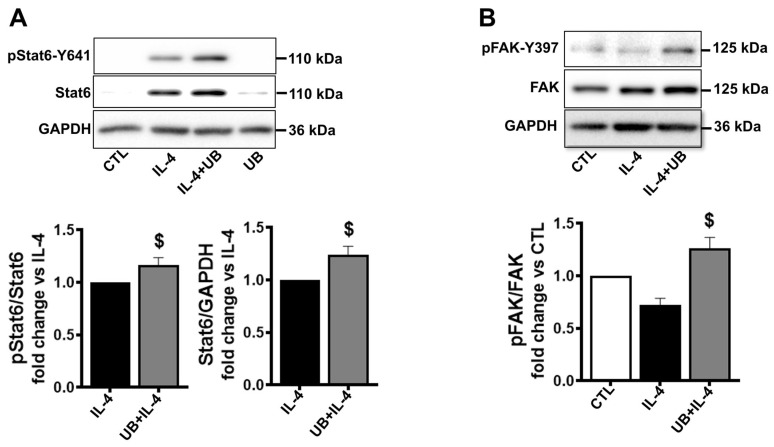
Exogenous UB affects STAT6 and FAK activation in M2-polarized macrophages. Peritoneal macrophages, pretreated with UB (20 µg/mL) for 30 min, were treated with IL-4 (20 ng/mL; M2 polarization) for 72 h. Cell lysates were analyzed by Western blot using anti-p-STAT6, STAT6, p-FAK, FAK, and GAPDH antibodies. After protein transfer, the membrane was cut into two portions. The upper portion, above the ~70 kDa marker, was probed for p-STAT6 or p-FAK, while the lower portion was probed for GAPDH. After stripping the upper portion, the membrane was probed for STAT6 or FAK. (**A**) The upper panel exhibits immunoblotting for p-STAT6, STAT6, and GAPDH. (**B**) The upper panel exhibits immunoblotting for p-FAK, FAK, and GAPDH. The lower panels (**A**,**B**) exhibit quantitative analyses normalized to STAT6, FAK, or GAPDH. Data were analyzed using a two-tailed Student’s *t* test or one-way ANOVA followed by a post hoc Tukey’s test. ^$^ *p* < 0.05 vs. IL-4; n = 3–6.

IL-4 is known to phosphorylate and activate STAT6. Activation of STAT6 is involved in the transcriptional control of many cytokines and chemokines [29]. Western blot analysis of cell lysates showed low-level expression of STAT6 in control samples and in those treated with eUB alone, with no detectable signal for p-STAT6. M2 polarization increased expression and phosphorylation of STAT6. However, expression and phosphorylation of STAT6 were significantly higher in M2-polarized cells treated with eUB versus IL-4 (Figure 6A). 

M2 polarization did not affect the expression or phosphorylation of FAK versus control. However, phosphorylation of FAK was significantly higher in M2-polarized cells treated with eUB versus IL-4 (Figure 6B). 

### 3.6. Exogenous UB Does Not Affect Ubiquitination of Cellular Proteins

**Figure 7:**
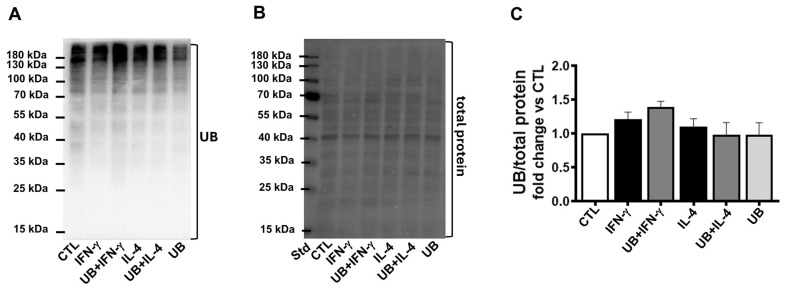
Exogenous UB had no effect on ubiquitination of cellular proteins. Peritoneal macrophages, pretreated with UB (20 µg/mL) for 30 min, were treated with IFN-γ (100 U/mL; M1 polarization) or IL-4 (20 ng/mL; M2 polarization) for 72 h. Cell lysates were analyzed by Western blot using anti-UB antibodies. (**A**) UB immunostaining. (**B**) Total protein stain. (**C**) Quantitative data for the ubiquitinated proteins normalized to total protein staining (entire lane). Data were analyzed using one-way ANOVA followed by a post hoc Tukey’s test. n = 3–4; Std, standard. The comparison groups were CTL, IFN-γ, UB+IFN-γ, and UB; and CTL, IL-4, UB+IL-4, and UB.

The primary intracellular function of UB is to covalently label proteins for degradation via the UPS [8,9]. Western blot analysis of cell lysates showed no significant change in the levels of ubiquitinylated proteins among the groups (Figure 7).

## 4. Discussion

Previous work has shown that eUB treatment reduces the number of infiltrates (neutrophils and macrophages) in the heart and is associated with cardioprotective effects in mice 3 days post-I/R [12]. eUB has also been shown to influence cell signaling and chemotaxis in THP-1 monocytes [13]. Additionally, it decreases mRNA markers of M1 polarization while increasing markers of M2 polarization in non-activated macrophages differentiated from THP-1 monocytes [14]. The data presented here provide evidence that eUB has immunomodulatory effects and affects the phenotype and function of M1- and M2-polarized macrophages. The major findings of this study are that eUB (1) decreases secretion of inflammatory cytokines in M1- and M2-polarized macrophages; (2) induces F-actin reorganization and decreases the cell surface area of M1-polarized macrophages; (3) decreases efferocytosis of M2-polarized macrophages; (4) enhances migration of M1-polarized macrophages; (5) decreases migration of M2-polarized macrophages; and (6) alters activation of signaling molecules (STAT1, STAT6, and FAK) in M1- and M2-polarized macrophages without affecting the ubiquitination of cellular proteins. In non-activated macrophages, eUB induces cell elongation, decreases secretory levels of pro-inflammatory cytokines/chemokines, and increases efferocytosis. 

Macrophages’ morphology is reported to modulate their phenotype and function [30]. Cellular elongation promotes M2 macrophage polarization and reduces the secretion of pro-inflammatory cytokines [30]. Conversely, preventing the spread of macrophages is associated with decreased M1 polarization and reduced inflammatory cytokine secretion [21]. In general, M1-polarized macrophages exhibit round and flattened morphology [20,30]. In non-activated macrophages differentiated from THP-1 monocytes, eUB treatment induced phenotypic alterations, resulting in cells becoming elongated, flat, and branching in comparison to the control [31]. Many of the functions of IFN-γ and IL-4, including regulation of the expression of cytokines and chemokines, are mediated via the activation of STAT1 and STAT6, respectively [26,27,29,32,33]. Here, M1-polarized macrophages exhibited round flattened morphology, while M2-polarized macrophages acquired elongated, spindle-shaped morphology. In M1-polarized macrophages, eUB induced F-actin reorganization and decreased the cell surface area, secretion of TNF-α, and expression and activation of STAT1. In M2-polarized macrophages, eUB decreased secretion of TNF-α and IL-10 and enhanced expression and activation of STAT6. Thus, eUB-mediated alterations in cell morphology and changes in STAT1 and STAT6 signaling may contribute to the decreased pro-inflammatory response observed in M1- and M2-polarized macrophages. Interestingly, eUB treatment in non-activated cells also induced cellular elongation and reduced the secretion of cytokines/chemokines (IL-1β, IL-2, and IL-5) without affecting STAT1 or STAT6. 

Following MI, neutrophils infiltrate the area of infarct and help to clear the wound of cellular debris before undergoing apoptosis [3,23]. Macrophages are the primary phagocytes responsible for clearing the apoptotic neutrophils and preventing secondary necrosis [34]. The effects of IFN-γ on macrophage efferocytosis and phagocytosis are reported to be variable. In human macrophages, IFN-γ had no effect on efferocytosis of apoptotic polymorphonuclear neutrophils [35], while it enhanced efferocytosis of apoptotic Jurkat cells and thymocytes in murine and human macrophages [36]. In this study, M1 polarization in the absence or presence of eUB had no effect on efferocytosis. Thus, the impact of IFN-γ on macrophage efferocytosis may depend on the apoptotic target cell [35]. Conversely, IL-4 treatment has been shown to increase efferocytosis of neutrophils and promote the resolution of the inflammatory phase post-MI in mice [37]. In immature granulocytes, eUB promoted phagocytosis of latex beads [38]. Consistent with these findings, we observed that M2 polarization and eUB treatment (in non-activated macrophages) enhanced efferocytosis compared to control. This increased efferocytosis with eUB in non-activated macrophages suggests a role for eUB in facilitating macrophage polarization towards M2 phenotype. Efferocytosis is crucial for maintaining tissue homeostasis and resolving inflammation [39]. eUB treatment decreased efferocytosis of M2-polarized macrophages. Therefore, the reduced efferocytosis in response to eUB in M2-polarized macrophages may serve as a regulatory mechanism to prevent excessive or premature clearance of inflammatory cells. 

The efflux of pro-inflammatory macrophages from infarcted myocardium may support inflammation resolution post-MI [24,25]. Macrophage migration in vivo occurs in the context of 2D and 3D environments [40]. Two-dimensional migration happens along extracellular matrix barriers like basement membranes or along inner vessel walls [40]. Saini et al. provided evidence that eUB serves as a chemotactic factor for THP-1 monocytic cells. In a filter migration assay, THP-1 cells migrated towards a concentration gradient of eUB [13]. M1 macrophages are reported to have increased adhesion to substrate and reduced cell migration [24]. Activation of FAK is involved in adhesion to the substrate, and dephosphorylation of FAK leads to the turnover of nascent adhesions, resulting in forward movement in cells [28]. The data presented here provide evidence that M1 polarization is associated with decreased 2D migration. eUB treatment enhanced 2D migration of M1-polarized macrophages. Activation of FAK was lower in M1-polarized macrophages treated with eUB. Therefore, the decreased activation of FAK (Tyr397) may help explain the increased migration observed in M1-polarized macrophages in the presence of eUB. Conversely, M2-polarized macrophages displayed enhanced migration and increased FAK activation. The enhanced migration of UB-treated M1-polarized macrophages may lead to their efflux from the infarct, potentially explaining the observed decrease in macrophage numbers in UB-treated hearts 3 days post-I/R [12]. On the other hand, the reduced migration of eUB-treated M2-polarized macrophages suggests that eUB may help maintain the presence of M2 macrophages longer in the infarct post-I/R. However, further investigations are needed to evaluate the effects of eUB on the efflux of M1- and M2-polarized macrophages post-I/R injury in vivo. 

## 5. Conclusions

The pro-inflammatory and anti-inflammatory phenotypes of macrophages are essential for wound healing post-MI, as they produce cytokines/chemokines and growth factors, and use phagocytosis to clear cellular debris and apoptotic cells [41]. In this study, eUB reduced cell size and the secretion of pro-inflammatory cytokines (TNF-α), enhanced cell migration, and modulated activation of STAT1 and FAK in M1-polarized macrophages. In M2-polarized macrophages, eUB reduced secretion of TNF-α and IL-10, efferocytosis, and migration, while increasing the activation of STAT6 and FAK. In non-activated macrophages, eUB induced cell elongation, decreased secretory levels of pro-inflammatory cytokines/chemokines, and increased efferocytosis, suggesting that eUB alone has the potential to polarize macrophages towards an anti-inflammatory (M2) phenotype. These effects of eUB occur without affecting the ubiquitination of cellular proteins. 

## 6. Limitations

The limitations of this study are as follows: a) macrophages display a range of phenotypes in vivo during the wound healing process [42], and multiple factors in the tissue microenvironment can influence their phenotype and function. In this study, M1 and M2 phenotypes were used to represent pro-inflammatory and anti-inflammatory phenotypes. b) The study used peritoneal macrophages differentiated into M1 and M2 phenotypes. Although these cells exhibit an inflammatory phenotype resulting from sterile inflammation, resembling the monocyte-derived macrophages in the heart post-I/R injury, further investigations are needed to fully understand the role of eUB in modulating macrophage phenotype and function in vivo in the heart following I/R injury. c) We recognize that while our findings achieved statistical significance, the sample size may be considered small. It should be noted that the number (n) represents biological replicates. Future work using macrophages isolated from the heart post-I/R injury with expanded sample sizes is needed to help validate these findings. d) The study outcomes were measured 72 h post-treatment, a timepoint chosen to polarize cells into M1 or M2 phenotypes. However, this selected timepoint may have overlooked temporal changes in cytokine secretion, signaling, or cell phenotype and function. A future time-course study may help determine whether the effects eUB are transient or sustained. e) The study used peritoneal macrophages isolated from male mice. To identify sex-differences, macrophages isolated from the peritoneal cavity of female mice should be analyzed to determine any sex-specific variations in phenotype and function.

## Data Availability

The original contributions presented in this study are included in the article/Supplementary Material. Further inquiries can be directed to the corresponding author.

## Supplementary Materials

The following supporting information can be downloaded at: https://www.mdpi.com/article/10.3390/cells14120879/s1, Figure S1: Western blot replicates of data presented in Figure 5A; Figure S2: Western blot replicates of data presented in Figure 5B; Figure S3: Western blot replicates of data presented in Figure 6A; Figure S4: Western blot replicates of data presented in Figure 6B; Figure S5: Western blot replicates of data presented in Figure 7; Figure S6: Western blot replicates of data presented in Figure 7. Table S1: Confidence Intervals and *p* values for Figure 1A–D; Table S2: Confidence Intervals and *p* values for Figure 1E–H; Table S3: Confidence Intervals and *p* values for Figure 2; Table S4: Confidence Intervals and *p* values for Figure 3; Table S5: Confidence Intervals and *p* values for Figure 4; Table S6: Confidence Intervals and *p* values for Figure 5; Table S7: Confidence Intervals and *p* values for Figure 6; Table S8: Confidence Intervals and *p* values for Figure 7.

## Abbreviations

The following abbreviations are used in this manuscript:

eUB	Exogenous ubiquitin.
I/R	Post-ischemia/reperfusion.
MI	Myocardial infarction.
M1	Pro-inflammatory macrophage.
M2	Anti-inflammatory macrophage.
UB	Ubiquitin.
UPS	Ubiquitin–proteasome system.
DMEM	Dulbecco’s modified Eagle’s medium.
FBS	Fetal bovine serum.
CTL	Control.
GM-CSF	Granulocyte–macrophage colony-stimulating factor.
IFN	Interferon.
TNF	Tumor necrosis factor.
PFA	Paraformaldehyde.
PMA	Phorbol-12-myristate-13-acetate.
ANOVA	One-way analysis of variance.
STAT1	Activate signal transducer and activator of transcription 1.
STAT6	Activate signal transducer and activator of transcription 6.
FAK	Focal adhesion kinase.
