# Supplementary material for: Exogenous Ubiquitin Differentially Modulates the Phenotype and Function of M1 and M2 Macrophages
Source: Cells. 2025 Jun 11;14(12):879. doi: 10.3390/cells14120879 (PMC12190236; doi:10.3390/cells14120879)
Supplement: Supplementary file 1 [file cells-14-00879-s001.zip › cells-3683072-supplementary.pdf]

**Figure S1. Western blot replicates of data presented in Figure 5A**

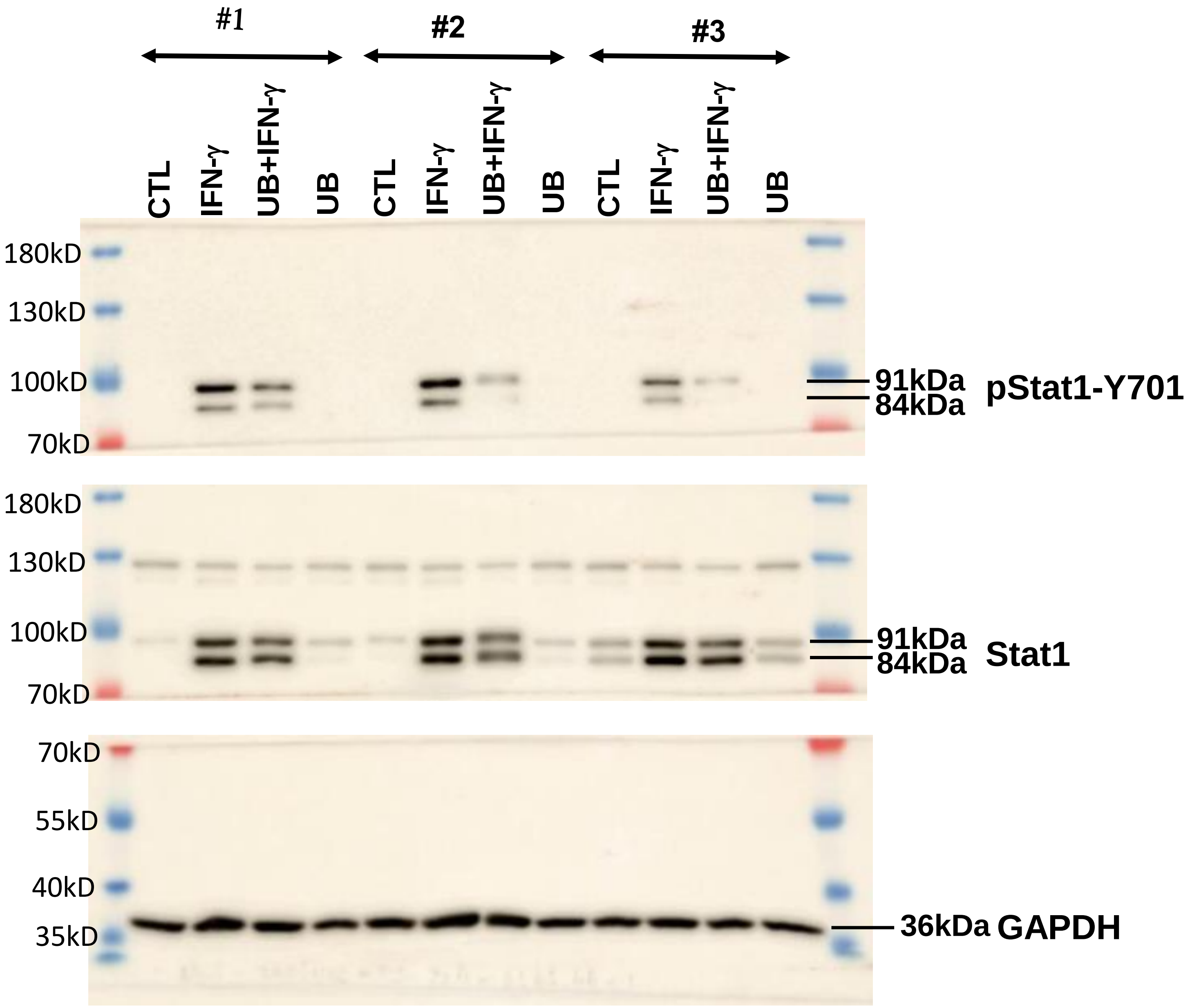

# Figure S2. Western blot replicates of data presented in Figure 5B

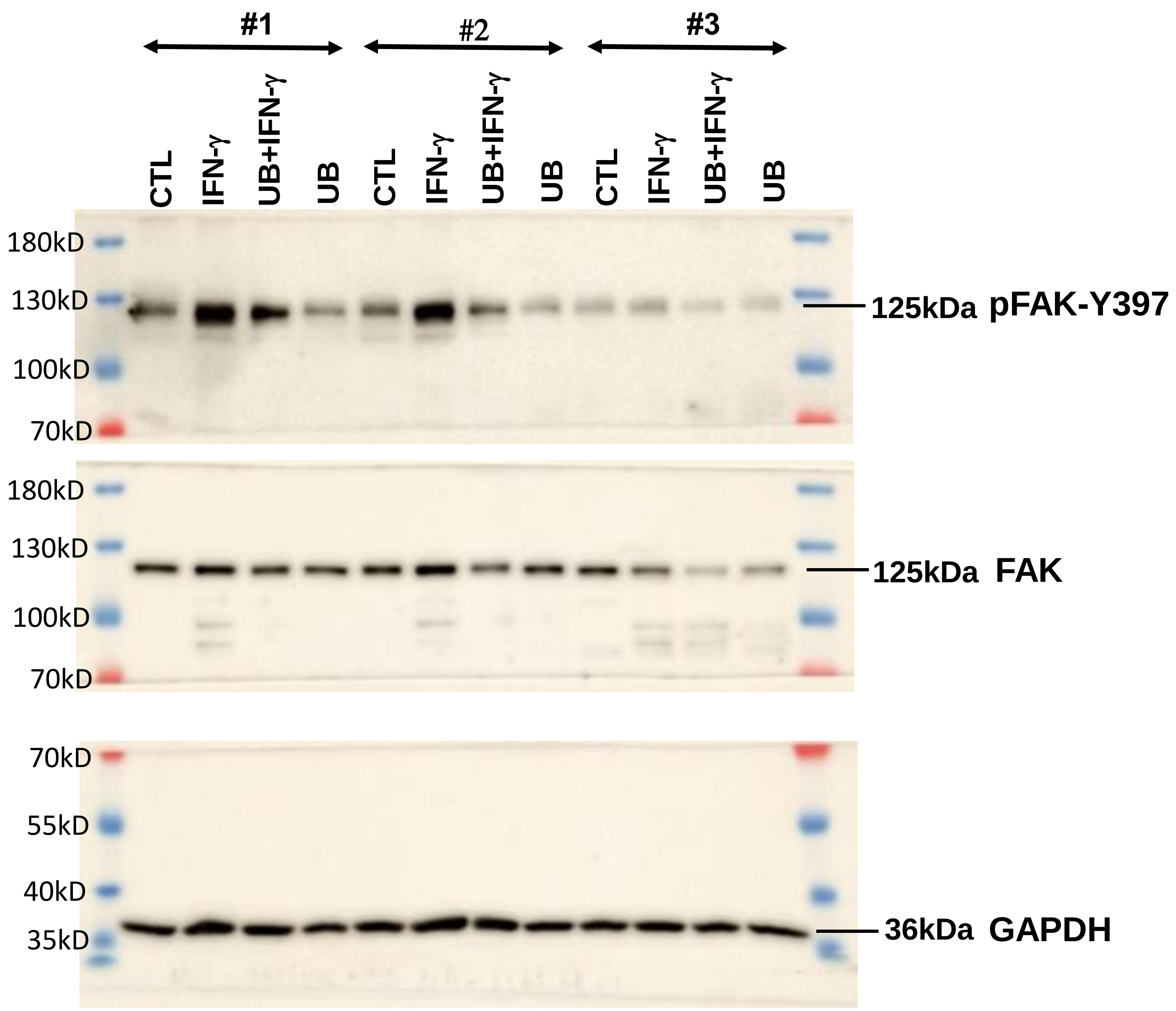

**Figure S3. Western blot replicates of data presented in Figure 6A**

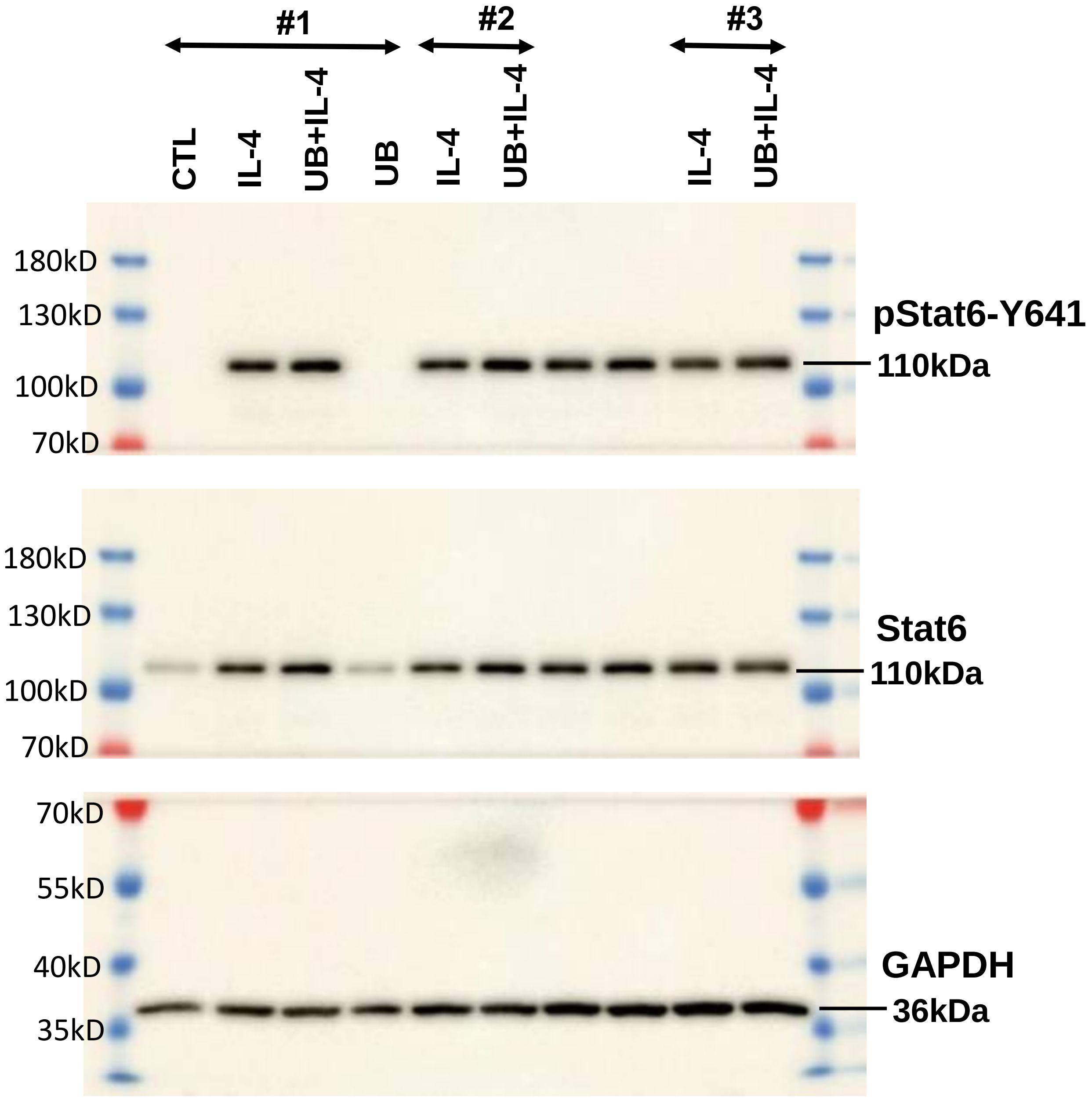


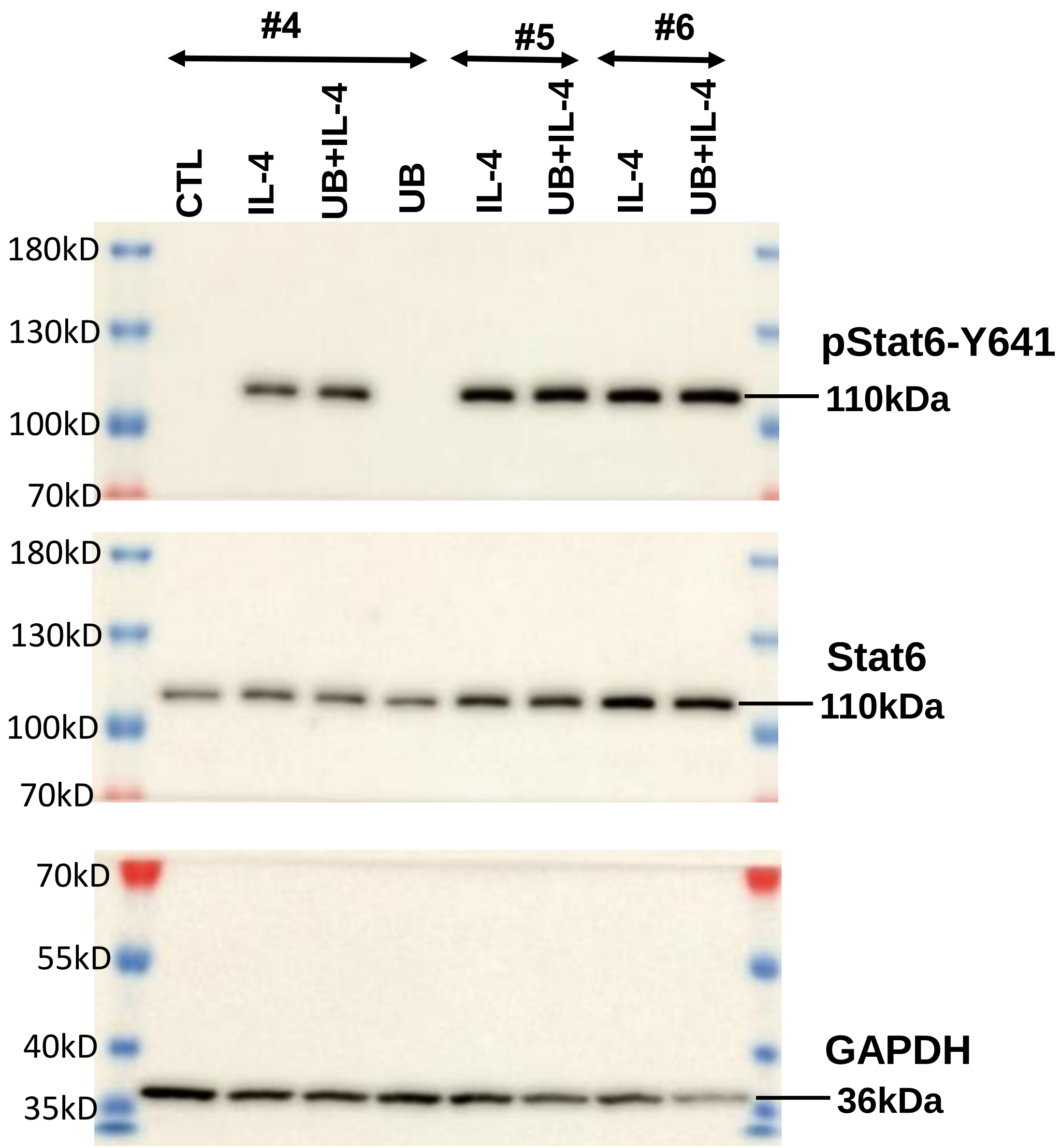

**Figure S4. Western blot replicates of data presented in Figure 6B**

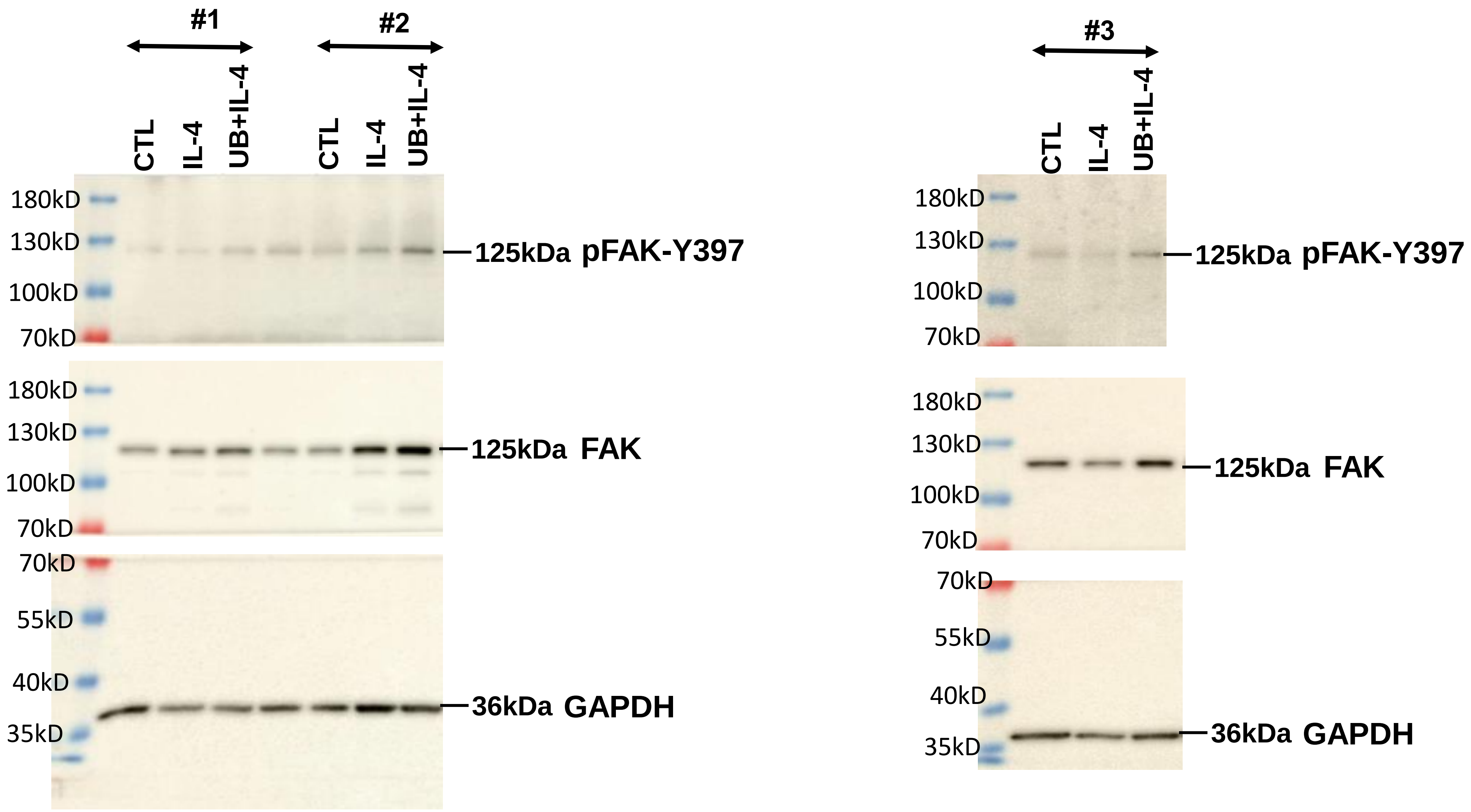

**Figure S5. Western blot replicates of data presented in Figure 7. Right hand panel shows membrane stained for total protein.**

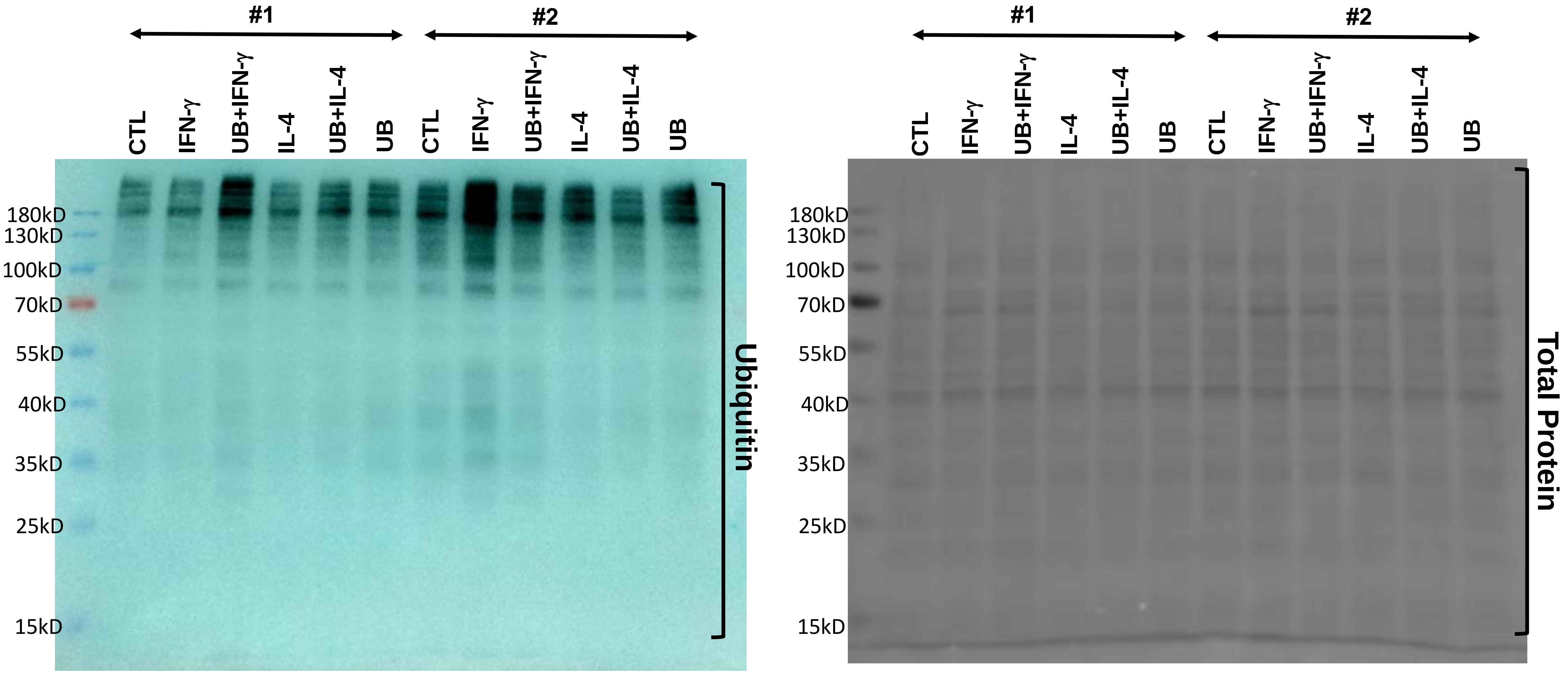

**Figure S6. Western blot replicates of data presented in Figure 7. Right hand panel shows membrane stained for total protein.**

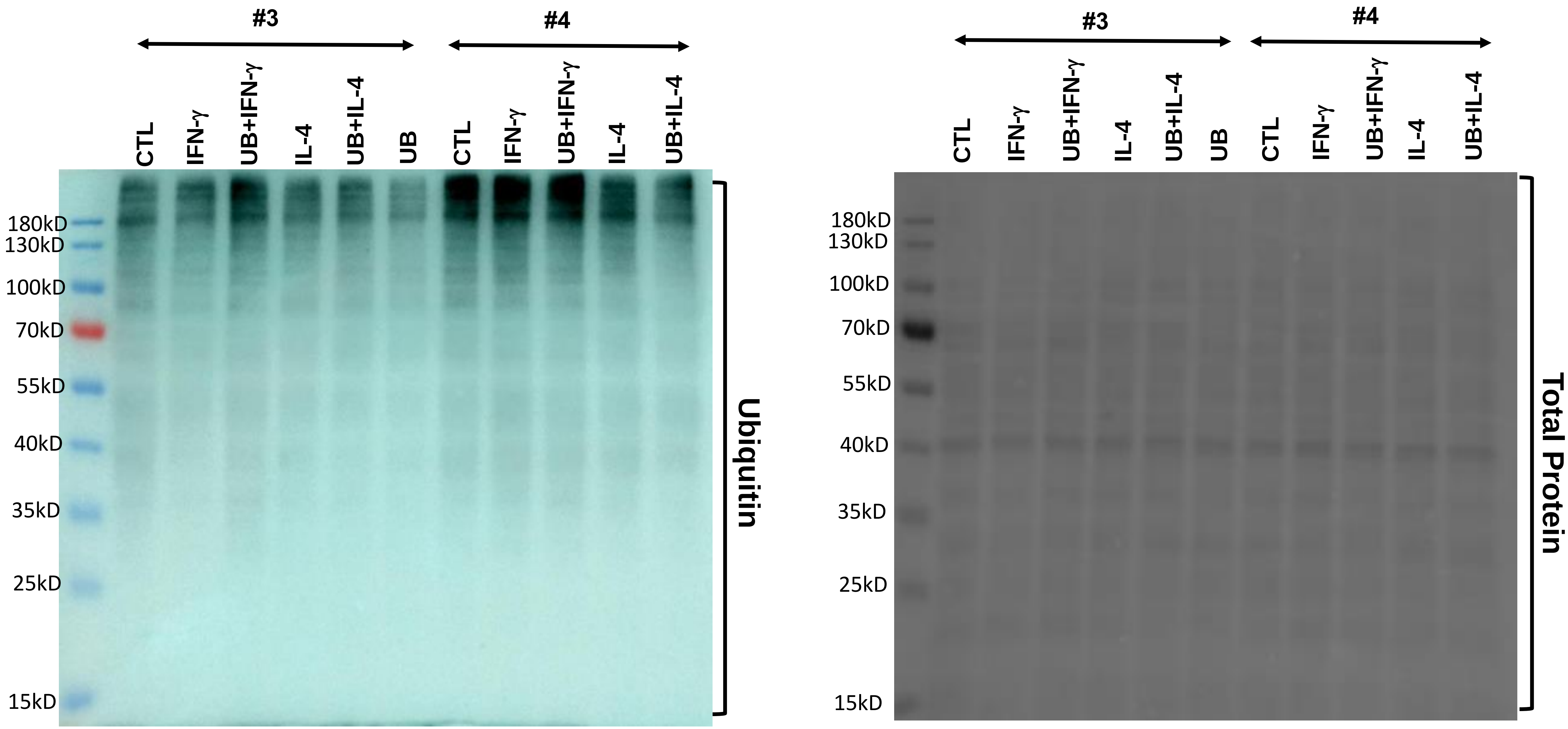

**Table S1. Confidence Intervals and p values for Figure 1 (A-D)**

| Cytokine | Groups | 95% CI of Diff. | p value |
|---|---|---|---|
| IFN-γ | CTL v IFN-γ | -51177 to -21004 | <0.0001 |
| | CTL v UB + IFN-γ | -46430 to -16258 | 0.0001 |
| | CTL v IL-4 | -69.56 to -53.33 | <0.0001 |
| | CTL v UB + IL-4 | -66.38 to -50.14 | <0.0001 |
| IL-4 | CTL v IL-4 | -48401 to -11720 | 0.0013 |
| | CTL v UB + IL-4 | 12320 to 17798 | <0.0001 (2-tailed TTest) |
| TNF-α | CTL v IFN-γ | -74.79 to -19.21 | 0.0009 |
| | IFN-γ v UB + IFN-γ | 3.246 to 58.83 | 0.0260 |
| | CTL v IL-4 | -92.82 to -36.02 | <0.0001 |
| | CTL v UB + IL-4 | -62.77 to -2.521 | 0.0316 |
| | IL-4 v UB + IL-4 | 1.651 to 61.90 | 0.0371 |
| IL-1β | CTL v IL-4 | -45.84 to -10.59 | 0.0016 |
| | CTL v UB + IL-4 | -44.39 to -9.131 | 0.0026 |
| | CTL v UB | 4.711 to 39.97 | 0.0110 |

**Table S2. Confidence Intervals and p values for Figure 1 (E-H)**

| Cytokine | Groups | 95% CI of Diff. | p value |
|---|---|---|---|
| IL-2 | CTL v IL-4 | -56.10 to -39.74 | <0.0001 |
| | CTL v UB + IL-4 | -51.15 to -34.79 | <0.0001 |
| | CTL v UB | 1.427 to 17.79 | 0.0187 |
| IL-5 | CTL v IL-4 | -1.066 to -0.2948 | 0.0006 |
| | CTL v UB + IL-4 | -1.117 to -0.3459 | 0.0003 |
| | CTL v UB | 0.09862 to 0.8700 | 0.0117 |
| IL-10 | CTL v IL-4 | -78.95 to -65.99 | <0.0001 |
| | CTL v UB + IL-4 | -71.44 to -58.48 | <0.0001 |
| | IL-4 v IL4 + UB | 1.029 to 13.99 | 0.0205 |
| GM-CSF | CTL v IL-4 | -82.79 to -65.50 | <0.0001 |
| | CTL v UB + IL-4 | -74.56 to -57.27 | <0.0001 |

**Table S3. Confidence Intervals and p values for Figure 2**

| Experimental variable | Groups | CI of difference | p value |
|---|---|---|---|
| Surface area | IFN-γ v UB + IFN-γ | -108.3 to -14.05 | 0.0227 (2-tailed TTest) |

**Table S4. Confidence Intervals and p values for Figure 3**

| Experimental variable | Groups | CI of difference | p value |
|---|---|---|---|
| Efferocytosis | CTL v IL-4 | -26.78 to -5.219 | 0.0063 |
| | IL-4 v IL-4 + UB | 2.719 to 24.28 | 0.0164 |
| | CTL v UB | -21.61 to -0.05261 | 0.0489 |

**Table S5. Confidence Intervals and p values for Figure 4**

| Experimental variable | Groups | CI of difference | p value |
|---|---|---|---|
| Wound healing | CTL v IFN-γ | 2.218 to 17.26 | 0.0055 |
| | CTL v IL-4 | -15.47 to -0.4245 | 0.0336 |
| | IFN-γ v UB + IFN-γ | -18.22 to -3.175 | 0.0020 |
| | IL-4 v UB + IL-4 | -10.40 to -0.5768 | 0.0328 (2-tailed TTest) |

**Table S6. Confidence Intervals and p values for Figure 5**

| Experimental variable | Groups | CI of difference | p value |
|---|---|---|---|
| pStat1 / Stat1 | IFN-γ v UB + IFN-γ | -1.036 to -0.1636 | 0.0188 (2-tailed TTest) |
| Stat1 / GAPDH | IFN-γ v UB + IFN-γ | -0.3470 to -0.05966 | 0.0171 (2-tailed TTest) |
| pFAK / FAK | IFN-γ v UB + IFN-γ | 0.06377 to 0.9896 | 0.0270 |
| | CTL v IFN-γ | -1.273 to -0.3471 | 0.0023 |

**Table S7. Confidence Intervals and p values for Figure 6**

| Experimental variable | Groups | CI of difference | p value |
|---|---|---|---|
| pStat6 / Stat6 | IL-4 v UB + IL-4 | 0.01779 to 0.3179 | 0.0319 (2-tailed TTest) |
| Stat6 / GAPDH | IL-4 v UB + IL-4 | 0.05203 to 0.4266 | 0.0173 (2-tailed TTest) |
| pFAK / FAK | IL-4 v UB + IL-4 | -0.8397 to -0.2343 | 0.0039 |

**Table S8. Confidence Intervals and p values for Figure 7 - no data were significant.**
